# Emergence of multidrug-resistant *Pandoraea sputorum* in Japan

**DOI:** 10.1128/mra.01166-23

**Published:** 2024-03-12

**Authors:** Juri Koizumi, Yutaka Nasu, Yuji Hirai, Hidemasa Nakaminami

**Affiliations:** 1 Department of Clinical Microbiology, School of Pharmacy, Tokyo University of Pharmacy and Life Sciences, Hachioji, Tokyo, Japan; 2 Department of Infectious Diseases, Tokyo Medical University Hachioji Medical Center, Hachioji, Tokyo, Japan; Department of Biological Sciences, Wellesley College, Wellesley, Massachusetts, USA

**Keywords:** *Pandoraea sputorum*, multidrug resistance, carbapenemase, antimicrobial resistance

## Abstract

*Pandoraea* is a pathogenic bacterium naturally resistant to various antimicrobials, including colistin. Here, we report the whole-genome sequence of *Pandoraea sputorum*, which exhibits high-level multidrug resistance, isolated from a hospitalized patient in Japan.

## ANNOUNCEMENT

*Pandoraea sputorum* is a gram-negative bacillus commonly isolated from patients with cystic fibrosis (CF) (1). *Pandoraea*, derived from the “Pandora’s box” (2), exhibit multidrug resistance (3). However, little is known about this bacterium. Here, we performed whole-genome sequencing of *P. sputorum*, having a high-level multidrug-resistant.

*P. sputorum* THI4931 was obtained from the sputum of an 84-year-old male patient at the Tokyo Medical University Hachioji Medical Center in 2019. The sputum sample was washed three times with sterilized saline and lysed with Sputazyme (Kyokuto Pharmaceutical, Japan). The lysate was plated on blood, BTB lactose, and chocolate agars (Nissui Pharmaceutical, Japan) and incubated at 37°C for 24 h under aerobic conditions. The grown colony species was identified using ID test NF-18 (Nissui Pharmaceutical) as an *Acinetobacter* species ID No. 773000. The strain was cultured aerobically on Mueller-Hinton agar (MHA: Becton Dickinson, USA) at 37℃ for 24 h and re-identified as *P. sputorum* by 16S rDNA sequencing (4). The antimicrobial susceptibility was evaluated by determining the minimum inhibitory concentration according to the Clinical and Laboratory Standards Institute method. As a breakpoint is not listed for *Pandoraea* species, it’s for *B. cepacia* were used. Genomic DNA was extracted from a single colony on MHA using phenol-chloroform-isoamyl alcohol (25:24:1) as previously described (5). Whole-genome sequencing was performed using Sequel IIe (Pacific Biosciences [PacBio], USA). g-TUBE (Covaris, USA) was used to shear the DNA fragments to approximately 10‒20 kbp. The SMRTbell Express template preparation kit v.2.0 (PacBio) was used to prepare the library; DNA size selection followed the manual. The resulting sequences, with the adapter sequences removed, were aligned using SMRT Link v.11.0.0.146107. Flye ver. 2.9.1-b1780 was used to assemble the reads. Bandage (ver. 0.8.1) and CheckM (ver. 1.2.2.) were used to check the results of the assembly and genome data, respectively. Prokka (ver. 1.14.5) and DDBJ Fast Annotation and Submission Tool (https://dfast.ddbj.nig.ac.jp) were used for annotation analysis. Basic Local Alignment Search Tool was used to compare amino acid and DNA sequences. Default parameters were used for all software unless otherwise specified.

Genome analysis revealed an N50 value of 5,833,454 bp, with 18,146 subreads. *P. sputorum* THI4931 had a 5,833,454 bp chromosome with 62.7% GC content (Accession no. AP028930) and no plasmid. *P. sputorum* THI4931 showed high-level resistance to some β-lactams, aminoglycosides, colistin, and polymyxin B (Table 1). In contrast, this strain was susceptible to aztreonam, imipenem, and minocycline. Two β-lactamases, AmpC and OXA-62 family carbapenemase, were found using ResFinder (http://genepi.food.dtu.dk/resfinder) and the Comprehensive Antibiotic Resistance Database (https://card.mcmaster.ca/), and the latter has been reported to be present in *Pandoraea* species (6). Previous reports have shown that *Pandoraea* species are resistant to antimicrobial agents (3, 7). *B. cepacia*, which is similar to *Pandoraea* species, is also naturally resistant to aminoglycosides and polypeptides, including colistin. The isolation of *P. sputorum* has been reported in China, England, Argentina, France, and Spain (1, 7–11); however, this is the first report of its isolation in Japan. *Pandoraea* species have been isolated from non-CF patients with underlying diseases (8); therefore, high-level multidrug-resistant *Pandoraea* species may pose a threat to hospitalized patients.

**TABLE 1:** Antimicrobial susceptibility of *Pandoraea sputorum* THI4931

Antimicrobial agent	MIC (μg/mL)	Antimicrobial agent	MIC (μg/mL)
Piperacillin	128	Piperacillin/tazobactam	16/2
Ceftazidime	≥256	Ceftriaxone	8
Cefdinir	32	Imipenem	2
Meropenem	64	Aztreonam	1
Ciprofloxaci	4	Levofloxacin	4
Clarithromycin	128	Clindamycin	≥256
Gentamicin	≥256	Amikacin	≥256
Minocyclin	0.25	Colistin	≥256
Polymyxin B	≥256		

## Data Availability

The genome sequence of the *P. sputorum* THI4391 chromosome was deposited in NCBI GenBank under the accession number AP028930. The DDBJ Sequence Read Archive (DRA) accession number for the raw reads is DRX488237.
